# Risk factors for acute kidney injury associated with intravenous vancomycin in neurosurgical inpatients: a retrospective study

**DOI:** 10.1007/s00228-023-03581-6

**Published:** 2023-10-27

**Authors:** Ruqi Lu, Junli Ren, Xuanping Zhou, Bin Zheng, Fangchen Peng

**Affiliations:** 1 School of Pharmacy, Shanxi Medical University, Taiyuan, Shanxi 030001 China; 2 Department of Pharmacy, Shanxi Provincial People’s Hospital, Shuangtasi Street 29#, Taiyuan, Shanxi 030012 China

**Keywords:** Vancomycin, Acute kidney injury, Neurosurgery, Mannitol, Loop diuretics

## Abstract

**Purpose:**

Vancomycin (VAN) is widely used in neurosurgical patients for intracranial infections. We aimed to assess the incidence and risk factors for VAN-associated acute kidney injury (VA-AKI) in this population.

**Methods:**

A case–control study of patients who treated with vancomycin in neurosurgery from January 2020 to December 2022 was conducted. Demographics and potential risk factors were collected. Multivariate logistic regression analyses were performed to identify risk factors for VA-AKI. AKI was defined according to the Kidney Disease Improving Global Outcomes Guidelines (KDIGO).

**Results:**

A total of 345 patients participated with a VA-AKI incidence of 17.1% (59 cases). Among them, 15 patients had renal impairment (Stage 2 or higher), and 2 required dialysis. With univariate analysis and binary logistic regression analysis, we found that the use of mannitol (OR: 4.164; 95% CI: 1.606–10.792; *P* = 0.003), loop diuretics (OR: 3.371; 95% CI: 1.633–6.958; *P* = 0.001), three or more antimicrobial applications (OR: 3.623; 95% CI: 1.600–8.206; *P* = 0.002), diastolic blood pressure 80–89 mm Hg (OR: 5.532; 95% CI: 1.677–18.250; *P* = 0.005) and diastolic blood pressure ≥ 90 mm Hg (OR: 6.845; 95% CI: 1.518–30.866; *P* = 0.012) were independent risk factors for VA-AKI. In addition, according to the Youden Index, the trough concentration of vancomycin should not exceed 15.845 mg/L.

**Conclusion:**

The incidence of VA-AKI in neurosurgical patients was 17.1%. The concomitant use of mannitol and loop diuretics, along with higher diastolic blood pressure and the combined use of more than three antimicrobial agents, were associated with an increased risk of neurosurgical VA-AKI.

## Introduction

Vancomycin (VAN) is a glycopeptide antibiotic primarily utilized to treat Gram-positive (G +) bacterial infections, particularly methicillin-resistant *Staphylococcus aureus* (MASA) and drug-resistant Enterococcus faecalis infections [1]. Due to its substantial blood–brain barrier penetration capability, VAN is considered a first-line empirical antibiotic for healthcare-associated infections in neurosurgical cases [2]. However, it has also been associated with severe acute kidney injury (AKI) [3, 4], a common clinical complication with a high risk of mortality and increased healthcare costs [5]. Previous reports have shown that the incidence of VAN-associated AKI (VA-AKI) varies widely among different patient groups, ranging from 5 to 43% [6]. A meta-study discovered that independent risk factors for VAN-related nephrotoxicity included VAN serum trough concentrations, treatment duration and intensive care unit (ICU) stay, and concomitant use of nephrotoxic medications (acyclovir, aminoglycosides, amphotericin B, loop diuretics, piperacillin-tazobactam and vasopressor medications) [7]. Diabetes mellitus, heart disease, liver disease, kidney disease and sepsis can also contribute to kidney damage [8].

Previous studies have primarily focused on critically ill patients in ICU when examining VAN-related nephrotoxicity [9, 10], with little known about surgical patients, especially those with neurosurgical VA-AKI. Compared with the general population, neurosurgical patients are often associated with increased intracranial pressure and disturbances in fluid and electrolyte balance, which may increase the risk of kidney injury. On the other hand, since neurosurgical patients are often exposed to surgical treatment, changes in postoperative physiopathologic status may affect the blood trough concentrations of VAN [2], which may affect the occurrence of AKI in patients. Nevertheless, no studies exists to analyze the incidence and risk factors for VA-AKI within neurosurgical patients. Therefore, this retrospective study aimed to assess the incidence and risk factors of VA-AKI in neurosurgical patients by collecting medical records of neurosurgical patients who received VAN, providing valuable insights for clinical application.

## Methods

### Study design

The sample for this retrospective case–control study was derived from individuals enrolled in the Department of Neurosurgery at Shanxi Provincial People's Hospital (Taiyuan, Shanxi Province, China). The hospital is a tertiary teaching hospital, with 2,500 beds, including 200 in neurosurgery. The study was approved by the medical ethics committee of Shanxi Provincial People's Hospital, China (No. 2023–247). Researchers waived the need to obtain written informed consent due to its retrospective nature. All data collected were kept confidential.

### Patients and data collection

**Fig. 1:**
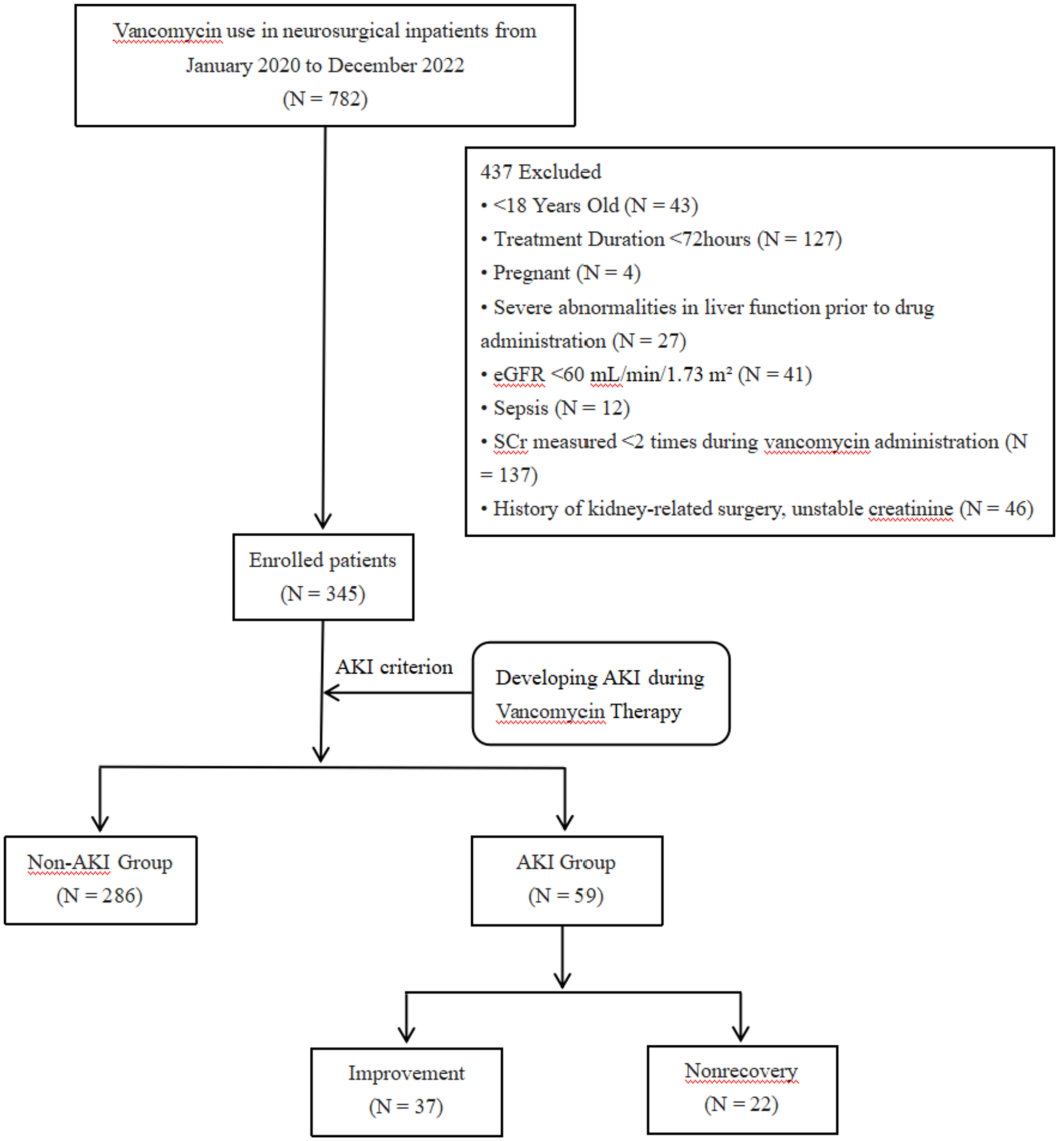
Study flow diagram. Abbreviations: AKI, acute kidney injury; eGFR, estimated glomerular filtration rate; SrCr, Serum creatinine upon admission

This retrospective case–control study included a total of 782 hospitalized patients who received VAN treatment from January 2020 to December 2022 in the Department of Neurosurgery at Shanxi Provincial People’s Hospital. We retrospectively analyzed 345 patients based on the following inclusion and exclusion criteria (Fig. 1). Inclusion criteria: age ≥ 18 years, treatment duration ≥ 72 h, no pre-existing renal disease (estimated glomerular filtration rate [eGFR] ≥ 60 mL/min/1.73 m^2^), stable serum creatinine (SCr) prior to VAN administration, and no history of renal-related surgery; exclusion criteria: severe hepatic insufficiency, pregnancy, sepsis, kidney surgery and patients without renal function indices or missing test results before and after treatment. Patients were divided into two groups for data analysis based on the development of nephrotoxicity (AKI Group) and those without nephrotoxicity (Non-AKl Group) to iden-tify risk factors associated with AKI development. The time of the first VAN administration was defined as the enrollment time, and the endpoint time was defined as the point patients presented with AKI, drug withdrawal, death, or discharge. The period between the enrollment time and the endpoint time was the followup period. In the AKI group, we further assessed the severity and outcome of the patients. According to the Kidney Disease Improving Global Outcomes Guidelines (KDIGO) criteria [11], patients observed were categorized based on the AKI staging (1, 2, or 3) to determine the severity of AKI. The outcome of AKI was determined by in-hospital death, the receipt of renal replacement therapy (RRT), renal improvement, and non-recovery at the time of hospital discharge. Kidney Improvement was defined as a decrease of at least 25% in the Scr level during hospitalization from the beginning of AKI onset; and non-recovery was defined as a lack of improvement in the Scr level at discharge.

The Ethics Committee of Shanxi Provincial People’s Hospital approved this study (No. 2023-247). Due to the retrospective nature of the study, written informed consent was waived by the researchers. Patient information was retrieved from the hospital’s electronic medical records and included age, sex, body mass index (BMI), SCr, serum urea value, eGFR, glutamine aminotransferase, alanine aminotransferase, albumin, infection status, duration of medication and duration of hospitalization. Additionally, data on nephrotoxic medications (see Table 1) and potential risk factors for nephrotoxicity (hypertension, diabetes mellitus, anemia, surgical history, systolic blood pressure and diastolic blood pressure) were collected.

**Table 1:** Baseline patient characteristics based on acute kidney injury during vancomycin therapy

Characteristics	Overrall	AKI	Non-AKI	*P*
	N = 345	N = 59 (17.1%)	N = 286 (82.9%)	
Male, n(%)	227 (65.8)	38 (64.4)	189 (66.1)	0.805
Age, years	56 (47–64)	54 (45–71)	56 (47–64)	0.696
BMI	23.67 (21.88–26.04)	23.67 (22.48–25.91)	23.65 (21.80–26.13)	0.902
Treatment goal				
Empirical	189 (54.8)	32 (54.2)	157 (54.9)	0.926
Intracranial infection	85 (24.6)	13 (22.0)	72 (25.2)	0.610
Inflammation of the lungs	52 (15.1)	10 (16.9)	42 (14.69)	0.658
Bloodstream infection	13 (3.8)	3 (5.1)	10 (3.5)	0.835
Skin and Soft Tissue Infections	6 (1.7)	1 (1.7)	5 (1.7)	1.000
Surgery	315 (91.3)	54 (91.5)	261 (91.3)	0.947
Comorbidities				
Diabetes	57 (16.5)	10 (16.9)	47 (16.4)	0.923
Hypertension	155 (44.9)	32 (54.2)	123 (43.0)	0.114
Anaemia	32 (9.3)	5 (8.5)	27 (9.4)	0.816
NICU	231 (67.0)	49 (83.1)	182 (63.6)	**0.004**
Hospitalization days, days	19 (15–24)	18 (15–23)	19 (15–24)	0.415
Vancomycin therapy duration, days	9 (6–13)	7 (5–14)	9.5 (6–12.25)	0.200
Monitoring trough concentrations	46 (13.3)	16 (27.1)	30 (10.5)	**0.001**
Number of combined antimicrobials	2 (2–2)	2 (2–3)	2 (1–2)	**< 0.001**
≥ 3 antimicrobials	39 (11.3)	17 (28.8)	22 (7.7)	**< 0.001**
Vancomycin monotherapy	81 (23.5)	9 (15.3)	72 (25.2)	0.102
Drug combination				
Piperacillin-tazobactam	26 (7.5)	6 (10.2)	20 (7.0)	0.400
Meropenem	149 (43.2)	31 (52.5)	118 (41.3)	0.111
Ceftriaxone	55 (15.9)	8 (13.6)	47 (16.4)	0.583
Cefoperazone-Sulbactam	23 (6.7)	5 (8.5)	18 (6.3)	0.541
Mannitol	234 (67.8)	53 (89.8)	181 (63.3)	**< 0.001**
Mannitol infusion rate^a^, g/kg/d	1.19 (1.03–1.58)	1.33 (1.14–1.99)	1.15 (0.99–1.47)	**0.001**
Loop diuretics	65 (18.8)	24 (40.7)	41 (14.3)	**< 0.001**
Diastolic blood pressure, mmHg	75 (69–80)	80 (76–86)	75 (68–78)	**< 0.001**
< 70	88 (25.50)	6 (10.17)	82 (28.67)	
70–79	165 (47.8)	23 (39.0)	142 (49.7)	
80–89	66 (19.1)	22 (37.3)	44 (15.4)	
≥ 90	26 (7.5)	8 (13.6)	18 (6.3)	
Systolic blood pressure, mmHg	127 (119–138.5)	136 (126–148)	126 (117.75–136.25)	**< 0.001**
< 120	89 (25.8)	7 (11.9)	82 (28.7)	
120–129	101 (29.3)	13 (22.0)	88 (30.8)	
130–139	73 (21.2)	16 (27.1)	57 (19.9)	
≥ 140	82 (23.8)	23 (39.0)	59 (20.6)	
eGFR, mL/min/1.73m2	104.38 (96.79–116.28)	102.66 (91.53–118.57)	104.67 (97.66–116.02)	0.400
Serum creatinine, umol/L	57.05 (48.12–67.18)	56.56 (44.24–73.26)	57.18 (48.82–66.50)	0.979
Serum urea, mmol/L	4.57 (3.39–6.03)	4.65 (3.23–6.13)	4.52 (3.45–6.03)	0.914
ALT, U/L	22.97 (14.20–43.44)	21.33 (13.31–41.47)	23.81 (14.34–45.30)	0.656
AST, U/L	26.40 (16.78–44.58)	25.96 (15.28–45.12)	26.46 (17.49–44.58)	0.738
Serum albumin, g/L	33.60 (30.40–37.00)	33.27 (29.06–36.57)	33.72 (30.43–37.05)	0.482

Data are presented as n (%) or median [IQR]. Variables with statistically significant differences are indicated in bold

*AKI* acute kidney injury, *BMI* body mass index, *NICU* neurosurgical intensive care unit, *ALT* alanine aminotransferase, *AST* glutamic oxaloacetic transaminase

^a^Only patients on mannitol were included, Mannitol infusion rate was obtained by dividing the cumulative dose of mannitol by body weight and duration of treatment

### AKI definition

We followed the diagnostic criteria for AKI outlined in the 2012 edition of the Kidney Disease: Improving Global Outcomes (KDIGO) Clinical Practice Guidelines [11]. AKI was defined as an increase in SCr of at least 0.3 mg/dL, or an increase in SCr of at least 1.5 times the baseline level within 48 h, or a sustained urine output of < 0.5 mL/(kg*h) for 6 h. Staging of AKI was performed according to the AKI staging criteria provided in the guidelines. Baseline creatinine was defined as the last recorded creatinine level before VAN administration. eGFR was calculated using the Chronic Kidney Disease Epidemiology Collaboration (CKD-EPI) equation, and an eGFR of less than 60 mL/min was used as the criterion for determining baseline renal impairment [12]. The CKD-EPI equation was as follows: GFR (in mL/min per 1.73 m^2^) = 141 × min (SCr/κ, 1) ^α × max (SCr/κ, 1) ^-1.209 × 0.993^age × 1.018 (if female) × 1.159 (if black). Here, SCr represented serum creatinine (in mg/dL), κ is 0.7 for females and 0.9 for males, and α was -0.329 for females and -0.411 for males.

### Statistical analysis

All statistical analyses were conducted using IBM SPSS 26.0 software (Armonk, NY, USA). Descriptive statistics were used to report the absolute and relative frequencies of categorical variables, while quantitative variables were presented as concentration trends and dispersion. The Mann–Whitney U test was employed to explore the correlation between AKI and each potential risk factor for non-normally distributed measures. Pearson’s chi-square test (χ^2^) or Fisher’s exact test was used for independent categorical variables. Logistic regression analysis was performed to identify the independent factors associated with VA-AKI. Variables found to be significantly associated with AKI in the univariate analysis were included in the binary logistic regression model. The goodness-of-fit was evaluated using the Homos-Lemeshow test, and the results were expressed as odds ratios (OR) with a 95% confidence interval (CI). Receiver operating characteristic (ROC) curve was used to investigated the nephrotoxicity threshold of different concentrations of VAN in serum. Additionally, an analysis is conducted on the duration of VAN administration and its co-administration with other medications. The Yoden index was used to determine the optimal threshold for nephrotoxicity. *P* < 0.05 was considered statistically significant.

## Results

A total of 782 patients received VAN treatment from January 2020 to December 2022. Among them, 437 patients were excluded, leaving a final sample of 345 patients. These patients were divided into two groups: the AKI group (N = 59) and the non-AKI group (N = 286), based on whether they developed AKI or not. Figure 1 illustrates the screening process. All enrolled patients received intravenous 1 g of VAN every 12 h, administered after mixing with sodium chloride injection or dextrose injection. Table 1 presents the baseline characteristics of the VAN-treated patients, showing no significant differences in sex distribution, age, BMI and type of infection between the AKI and non-AKI groups. The median age of the patients was 56 years (interquartile range: 47–64), with 227 (65.8%) males and 118 (34.2%) females. Among the enrolled patients, 231 (67.0%) were admitted to the Neurosurgical Intensive Care Unit (NICU), with a median duration of VAN treatment of 9 days and a median length of hospitalization of 19 days.

The primary indication for VAN treatment is intracranial infection. In most cases, the treatment was empirical. Combination therapy with antibiotics was common in both groups. Among the AKI group, patients receiving piperacillin-tazobactam and meropenem were more prevalent, but the difference was not statistically significant (10.2% vs 7.0%, p = 0.400; 52.5% vs 41.3%, *P* = 0.111). Monotherapy with VAN for infections did not appear to impact the occurrence of nephrotoxicity (*P* = 0.102); however, the number of antibiotics used appears to be associated with nephrotoxicity (*P* < 0.001). We categorized the number of antibiotics and found that the proportion of nephrotoxicity was significantly higher in patients using three or more antibiotics than in those with less than three (28.8% vs. 7.7%, *P* < 0.001) (Table 1).

In the logistic regression analysis, we incorporated several variables such as NICU admission, use of three or more antibiotics, use of mannitol, use of loop diuretics, diastolic and systolic blood pressure. The results found that patients using three or more antibiotics had a higher risk of developing AKI compared to those using fewer than three antibiotics (OR: 3.623, 95% CI: 1.600–8.206, *P* = 0.002). Patients using mannitol (OR: 4.164, 95% CI: 1.606–10.792, *P* = 0.003) or loop diuretics (OR: 3.371, 95% CI: 1.633–6.958, *P* = 0.001) also exhibited an elevated risk of AKI. Diastolic blood pressure levels of 80–89 mm Hg (OR: 5.532, 95% CI: 1.677–18.250, *P* = 0.005) and diastolic blood pressure ≥ 90 mm Hg (OR: 6.845, 95% CI: 1.518–30.866, *P* = 0.012) were also identified as independent risk factors for AKI (Table 2).

**Table 2:** Regression analysis of risk factors for vancomycin-associated acute kidney injury in neurosurgery

	Univariate analysis			Multivariate analysis		
	OR	95% CI	*P*	OR	95% CI	*P*
Age > 60 years	1.065	0.602–1.886	0.828			
Hypertension	1.571	0.894–2.758	0.116			
NICU	2.800	1.361–5.761	**0.005**	1.011	0.428–2.389	0.979
≥ 3 antimicrobials	4.857	2.384–9.897	**< 0.001**	3.623	1.600–8.206	**0.002**
Vancomycin monotherapy	0.535	0.251–1.142	0.106			
Piperacillin-tazobactam	1.506	0.577–3.927	0.403			
Meropenem	1.576	0.898–2.767	0.113			
Mannitol	5.124	2.130–12.327	**< 0.001**	4.164	1.606–10.792	**0.003**
Loop diuretics	4.098	2.214–7.585	**< 0.001**	3.371	1.633–6.958	**0.001**
Diastolic blood pressure						
< 70	1.000			1.000		
70–79	2.214	0.866–5.659	0.097	2.071	0.707–6.066	0.184
80–89	6.833	2.580–18.101	**< 0.001**	5.532	1.677–18.250	**0.005**
≥ 90	6.074	1.876–19.669	**0.003**	6.845	1.518–30.866	**0.012**
Systolic blood pressure						
< 120	1.000			1.000		
120–129	1.731	0.658–4.551	0.266	1.049	0.338–3.250	0.934
130–139	3.288	1.271–8.505	**0.014**	1.331	0.417–4.242	0.629
≥ 140	4.567	1.839–11.343	**0.001**	1.504	0.452–5.012	0.506

Variables with statistically significant differences are indicated in bold

*NICU* neurosurgical intensive care unit, *OR* odd ratio, *95% CI* 95% confidence interval

Table 3 summarizes the clinical presentation and outcomes of patients with VA-AKI. In terms of AKI severity, the majority of patients (74.6%) were classified as KDIGO stage 1, among whom only 12 patients underwent medication discontinuation. Among all AKI cases, 49 patients (83.1%) developed AKI within 1 week, and 32 patients (62.7%) achieved complete recovery with a return of SCr to baseline levels. Renal replacement therapy was required for two of the AKI patients (3.4%), and there were four deaths (6.8%); however, the cause of death was not directly related to renal injury.

**Table 3:** Outcome of 59 patients with vancomycin-associated acute kidney injury^a^

Outcome		AKI Stages		Discontinuation^b^	Onset of Acute Kidney Injury	
Recovery	37 (62.7)	Stage 1	44 (74.6)	12 (27.3)	≤ 3 days	30 (50.8)
Do not recover	16 (27.1)	Stage 2	11 (18.6)	6 (54.5)	4–7 days	19 (32.2)
RRT	2 (3.4)	Stage 3	4 (6.8)	2 (50.0)	≥ 7 days	10 (16.9)
Death^c^	4 (6.8)	——	——		——	——

*AKI* acute kidney injury, *RRT* renal replacement therapy

^a^Data are expressed as number (percentage) unless specified

^b^The number of individuals discontinuing vancomycin in each stage

^c^None of these patients died because of vancomycin-related acute kidney injury

**Fig. 2:**
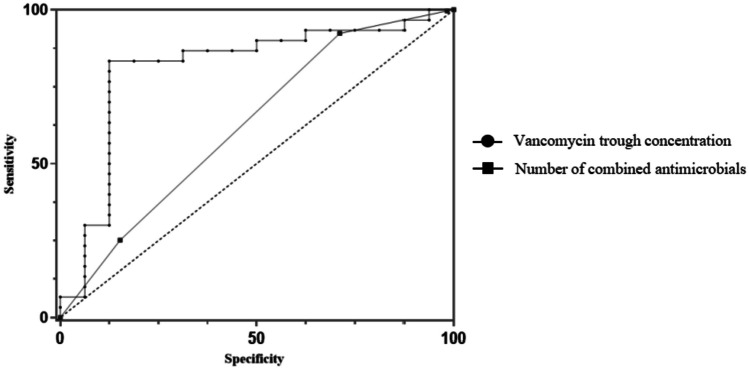
Receiver Operating Characteristic (ROC) curve

In this study, there were only 46 patients were monitored for trough concentrations after the fourth dose. Based on data from these 46 patients, we constructed ROC curves and employed the Youden index to analyze the thresholds (Fig. 2). The area under the ROC curves for VAN trough concentration and the number of antibiotics were 0.810 and 0.625, with corresponding thresholds of 15.845 and 2.5 (Table 4).

**Table 4:** Results of ROC

Variable	AUC	P	Threshold value
Vancomycin trough concentration, mg/L	0.810	0.001	15.845
Number of combined antimicrobials, n	0.625	0.003	2.5

*ROC* receiver operating characteristic, *AUC* areas under the curve

## Discussion

To the best of our knowledge, this is the first report about AKI induced by VAN in neurosurgical patients. In this study, we found that combined use of mannitol, loop diuretics, number of antibiotic use and elevated diastolic blood pressure were associated with increased risk of AKI.

Very few studies have explored the relationship between mannitol and VAN. Mannitol was not found to be a risk factor for VA-AKI in the study by Pan et al. [13], which is contrary to our findings. This discrepancy may stem from the patient population in our study, as our subjects were all from neurosurgery, and mannitol is widely used in this field to alleviate cerebral edema, which undoubtedly reduces the body’s blood volume, thus further increasing the potential risk of AKI. In our study, patients treated with mannitol demonstrated a 4.164-fold increase in the odds of AKI. Although the mechanism underlying the use of mannitol and the development of VA-AKI has not been fully elucidated, potential reasons include renal vasoconstriction induced by high doses of mannitol, as well as mannitol-induced swelling and vacuolization of proximal tubules, which undoubtedly exacerbate the deterioration of renal function [14]. Mannitol is not metabolized in the body and is excreted via glomerular filtration. In patients with renal impairment, the half-life of mannitol can increase from 2–4 h to > 36 h [14, 15], suggesting a possible cumulative effect that contributes to more severe AKI. Because mannitol was not administered to some patients in our study, it is difficult to draw definitive conclusions regarding the clinical correlation between mannitol dose and VA-AKI. Further studies are needed to explore more clearly the relationship between mannitol dose and renal function in patients treated with VAN. In Table 1, we excluded patients who did not receive mannitol, allowing for further analysis of the effect of mannitol infusion rate on VA-AKI. The study found that the AKI group had a significantly higher mannitol infusion rate compared to the control group, indirectly suggesting that a high daily dose of mannitol was more likely to increase the risk of VA-AKI, potentially occurring at a certain threshold value [11]. Therefore, in the clinical application of VAN therapy, physicians should avoid high-dose mannitol infusion to minimize potential risk of renal injury.

The use of loop diuretics also significantly increases the risk of VA-AKI, consistent with previous findings [7]. Prerenal factors lead to inadequate renal perfusion by decreasing the body's effective circulating blood volume, consequently resulting in AKI [16–18]. Loop diuretics function by inhibiting the reabsorption of sodium ions in the thick ascending limb of the renal tubules, thereby raising cardiac filling pressure and reducing fluid retention within the body. However, due to various reasons, such as the activation of the renin-angiotensin system or the sympathetic nervous system, loop diuretics can also potentially lead to a decrease in overall blood volume and potential renal hypoperfusion, further reducing renal function [19, 20].

Several studies have demonstrated an increased risk of AKI when VAN was combined with piperacillin tazobactam [21, 22]. However, our study did not find this correlation, possibly because the common antimicrobial drug combinations used in our neurosurgery department for patients receiving VAN were meropenem or ceftriaxone, which are not typically associated with an increased risk of VA-AKI [23–25]. Nonetheless, we did observe a higher proportion of patients in the AKI group who received a combination of meropenem or piperacillin tazobactam. Interestingly, although the antibiotic combinations we considered did not show statistical differences, the number of antibiotics used appeared to have a positive correlation with the incidence of VA-AKI. Specifically, the use of three or more antibiotics increased the risk of VA-AKI by 3.623-fold. This may be explained by the more severe physiopathological state of patients who received three or more antibiotics, which in turn increased the risk of VA-AKI. Therefore, we recommend that clinical physicians monitor the renal function more closely in patients receiving multiple antibiotic treatments to address the potential occurrence of AKI. Lee et al. [26] found that higher systolic and diastolic blood pressure levels were associated with an increased risk of worsening renal function in a large prospective cohort study. In our study, we investigated the effects of systolic and diastolic blood pressure on VA-AKI and found that patients who developed VA-AKI had higher systolic and diastolic blood pressure. Regression analysis showed that higher diastolic blood pressure was an important risk factor for the development of AKI in VAN-treated patients. Additionally, a history of hypertension was more common in patients with VA-AKI, and the correlation between higher diastolic blood pressure and VA-AKI could be explained by more severe or uncontrolled hypertension.

Among the patients monitored the trough concentration, the proportion of patients with AKI was significantly higher than that in the control group. It is worth noting that VAN Therapeutic drug monitoring (TDM) started late in our hospital, and clinicians often tend to select patients with severe conditions for trough concentration monitoring when assessing their conditions, which explains why the proportion of AKI was higher in patients who were monitored for trough concentration. In this regard, it is necessary for clinicians to deepen their understanding of the importance of VAN concentration monitoring. A retrospective study showed that VAN nephrotoxicity was associated with trough concentration levels, with the risk of AKI being 1.36 and 1.76 times higher in patients with trough concentrations between 15–20 mg/L and > 20 mg/L than in the control group (< 10 mg/L) [27]. Based on statistical trough concentration data, we simulated an ROC curve, and according to the Youden index, the trough concentration of VAN should not exceed 15.845 mg/L.

There were several limitations to this study. First, it was a retrospective analysis, and patients with missing data were excluded from the analysis. Moreover, the number of SCr measurements per patient was inconsistent, making it challenging to accurately evaluate VAN nephrotoxicity. Third, due to the unavailability of the Glasgow Coma Score, we were unable to assess the relationship between illness severity and AKI.

## Conclusion

This is the first report about AKI induced by VAN in neurosurgical patients. The results showed that the incidence of VA-AKI in neurosurgical patients was 17.1%. Concomitant application of mannitol and loop diuretics significantly increased the risk of AKI, and higher mannitol doses and infusion rates may be associated with more frequent and more severe AKI. In addition, higher diastolic blood pressure and the combination of more than three antibiotics were also associated with the development of AKI. It is recommended that VAN monitoring trough concentrations should not exceed 15 mg/L, and more renal function monitoring should be employed in patients receiving multiple antibiotic therapy to prevent more severe kidney injury. Considering the low rate of TDM monitoring, clinicians should always monitor SCr levels or estimate GFR to prevent AKI.

## Data Availability

The datasets generated during the current study are available from the corresponding author on reasonable request.

## References

[1] Sy CL, Chen PY, Cheng CW, Huang LJ, Wang CH, Chang TH, Chang YC, Chang CJ, Hii IM, Hsu YL, Hu YL, Hung PL, Kuo CY, Lin PC, Liu PY, Lo CL, Lo SH, Ting PJ, Tseng CF, Wang HW, Yang CH, Lee SS, Chen YS, Liu YC, Wang FD (2022). Recommendations and guidelines for the treatment of infections due to multidrug resistant organisms. J Microbiol Immunol Infect.

[2] Kim AJ, Lee JY, Choi SA, Shin WG (2016). Comparison of the pharmacokinetics of vancomycin in neurosurgical and non-neurosurgical patients. Int J Antimicrob Agents.

[3] Liu C, Bayer A, Cosgrove SE, Daum RS, Fridkin SK, Gorwitz RJ, Kaplan SL, Karchmer AW, Levine DP, Murray BE, Rybak MJ, Talan DA, Chambers HF (2011). Clinical practice guidelines by the infectious diseases society of america for the treatment of methicillin-resistant Staphylococcus aureus infections in adults and children: executive summary. Clin Infect Dis.

[4] Sinha Ray A, Haikal A, Hammoud KA, Yu ASL (2016). Vancomycin and the risk of AKI: a systematic review and meta-analysis. Clin J Am Soc Nephrol.

[5] Huang J, Xu D, Yang L (2020). Acute kidney injury in Asia: disease burden. Semin Nephrol.

[6] van Hal SJ, Paterson DL, Lodise TP (2013). Systematic review and meta-analysis of vancomycin-induced nephrotoxicity associated with dosing schedules that maintain troughs between 15 and 20 milligrams per liter. Antimicrob Agents Chemother.

[7] Kim JY, Yee J, Yoon HY, Han JM, Gwak HS (2022). Risk factors for vancomycin-associated acute kidney injury: a systematic review and meta-analysis. Br J Clin Pharmacol.

[8] James MT, Grams ME, Woodward M, Elley CR, Green JA, Wheeler DC, de Jong P, Gansevoort RT, Levey AS, Warnock DG, Sarnak MJ (2015). A meta-analysis of the association of estimated GFR, albuminuria, diabetes mellitus, and hypertension with acute kidney injury. Am J Kidney Dis.

[9] Perin N, Roger C, Marin G, Molinari N, Evrard A, Lavigne JP, Barbar S, Claret PG, Boutin C, Muller L, Lipman J, Lefrant JY, Jaber S, Roberts JA (2020). Vancomycin serum concentration after 48 h of administration: a 3-years survey in an intensive care unit. Antibiotics (Basel).

[10] Fuhrman DY, Kane-Gill S, Goldstein SL, Priyanka P, Kellum JA (2018). Acute kidney injury epidemiology, risk factors, and outcomes in critically ill patients 16–25 years of age treated in an adult intensive care unit. Ann Intensive Care.

[11] Kim MY, Park JH, Kang NR, Jang HR, Lee JE, Huh W, Kim YG, Kim DJ, Hong SC, Kim JS, Oh HY (2014). Increased risk of acute kidney injury associated with higher infusion rate of mannitol in patients with intracranial hemorrhage. J Neurosurg.

[12] Hoste L, Dubourg L, Selistre L, De Souza VC, Ranchin B, Hadj-Aïssa A, Cochat P, Martens F, Pottel H (2014). A new equation to estimate the glomerular filtration rate in children, adolescents and young adults. Nephrol Dial Transplant.

[13] Pan KM, Wu Y, Chen C, Chen ZZ, Xu JA, Cao L, Xu Q, Wu W, Dai PF, Li XY, Lv QZ (2018). Vancomycin-induced acute kidney injury in elderly Chinese patients: a single-centre cross-sectional study. Br J Clin Pharmacol.

[14] Purnomo AF, Permana KR, Daryanto B (2021). Acute kidney injury following mannitol administration in traumatic brain injury: a meta-analysis. Acta Inform Med.

[15] Nau R (2000). Osmotherapy for elevated intracranial pressure: a critical reappraisal. Clin Pharmacokinet.

[16] Huang M, Wu H, Zhou J, Xu M, Zhou S (2018). Efficacy of vancomycin on gram-positive bacterial infection in elderly critical patients and risk factors associated with nephrotoxicity. Arch Iran Med.

[17] Perner A, Prowle J, Joannidis M, Young P, Hjortrup PB, Pettilä V (2017). Fluid management in acute kidney injury. Intensive Care Med.

[18] Epstein FH, Badr KF, Ichikawa I (1988). Prerenal failure: a deleterious shift from renal compensation to decompensation. N Engl J Med.

[19] Dikshit K, Vyden JK, Forrester JS, Chatterjee K, Prakash R, Swan HJ (1973). Renal and extrarenal hemodynamic effects of furosemide in congestive heart failure after acute myocardial infarction. N Engl J Med.

[20] Leete J, Wang C, López-Hernández FJ, Layton AT (2022). Determining risk factors for triple whammy acute kidney injury. Math Biosci.

[21] Tookhi RF, Kabli NA, Huntul MA, Thabit AK (2021). Impact of combining vancomycin with piperacillin/tazobactam or with meropenem on vancomycin-induced nephrotoxicity. Intern Emerg Med.

[22] Wali HA, Alabdulwahed MA, Al-Hussain GY, Alabbad MA, Al-Khalaf LH, Alhumaidi SS, Alhussain K, Alomair SM, Almulhim AS (2023). Assessment of knowledge, attitude, and practices of acute kidney injury incidence with co-administration of piperacillin/tazobactam and vancomycin among healthcare workers: a cross-sectional study. Saudi Pharm J.

[23] Zhang T, Cheng H, Li Y, Dong YZ, Zhang Y, Cheng XL, Wang AM, Dong YL (2019). Paediatric acute kidney injury induced by vancomycin monotherapy versus combined vancomycin and meropenem. J Clin Pharm Ther.

[24] Chen AY, Deng CY, Calvachi-Prieto P, Armengol de la Hoz MÁ, Khazi-Syed A, Chen C, Scurlock C, Becker CD, Johnson AEW, Celi LA, Dagan A (2023). A large-scale multicenter retrospective study on nephrotoxicity associated with empiric broad-spectrum antibiotics in critically ill patients. Chest.

[25] Moon YS, Chung KC, Gill MA (1997). Pharmacokinetics of meropenem in animals, healthy volunteers, and patients. Clin Infect Dis.

[26] Lee JY, Park JT, Joo YS, Lee C, Yun HR, Yoo TH, Kang SW, Choi KH, Ahn C, Oh KH, Sung S, Kim SW, Lee J, Han SH, KNOW-CKD (Korean Cohort Study for Outcomes in Patients With Chronic Kidney Disease) Investigators (2021). Association of blood pressure with the progression of CKD: findings from KNOW-CKD study. Am J Kidney Dis.

[27] Li L, Zhang L, Li S, Xu F, Li L, Li S, Lyu J, Yin H (2022). Effect of first trough vancomycin concentration on the occurrence of AKI in critically Ill patients: a retrospective study of the MIMIC-IV database. Front Med (Lausanne).

